# Associations between human leukocyte antigen polymorphisms and hypersensitivity to antiretroviral therapy in patients with human immunodeficiency virus: a meta-analysis

**DOI:** 10.1186/s12879-019-4227-5

**Published:** 2019-07-05

**Authors:** Kun Hu, Qian Xiang, Zhe Wang, Guang-yan Mu, Zhuo Zhang, Ling-yue Ma, Qiu-fen Xie, Shu-qing Chen, Shuang Zhou, Xiao-dan Zhang, Yi-min Cui

**Affiliations:** 1 Department of Pharmacy, Peking University First Hospital, 6# Dahongluochang Street, Xicheng District, Beijing, China; 2 Department of Pharmacy Administration and Clinical Pharmacy, School of Pharmacy, Peking University Health Science Center, 38# Xueyuan Road, Haidian District, Beijing, China

**Keywords:** HLA, Polymorphisms, Hypersensitivity, Antiretroviral therapy, HIV

## Abstract

**Background:**

Human leukocyte antigen (HLA) alleles are implicated in drug-induced hypersensitivity, including by nevirapine and abacavir. The purpose of this meta-analysis was to evaluate the relationship between HLA polymorphisms and hypersensitivity to antiretroviral therapy in human immunodeficiency virus (HIV)-infected patients.

**Methods:**

We conducted a systematic search of PubMed, Embase, Web of Science, and the Cochrane Library for studies that evaluated the associations of HLA polymorphisms with antiretroviral therapy-induced hypersensitivity published in April 2019. The summary odds ratios (ORs) with 95% confidence intervals (CIs) were considered as estimates of the effect.

**Results:**

The meta-analysis included 17 studies that assessed a total of 4273 patients. First, carriers of HLA-A *24 were associated with an increased risk of hypersensitivity among patients with HIV who received antiretroviral therapy (OR: 12.12; *P* = 0.018). Second, five SNPs of HLA-B genotypes, including *18 (OR: 1.63; *P* = 0.028), *35 (OR: 2.31; *P* = 0.002), *39 (OR: 11.85; *P* = 0.040), *51 (OR: 1.66; *P* = 0.028), and *81 (OR: 8.11; *P* = 0.021), were associated with an increased risk of hypersensitivity. Conversely, carriers of HLA-B *15 were associated with a reduced risk of hypersensitivity (OR: 0.43; *P* < 0.001). Third, HLA-C *04 was associated with an increased risk of hypersensitivity (OR: 3.09; *P* < 0.001), whereas a lower risk for hypersensitivity was observed in patients who were carriers of HLA-C *02 (OR: 0.22; *P* = 0.030), *03 (OR: 0.53; *P* = 0.049), and *07 (OR: 0.61; *P* = 0.044). Finally, carriers of HLA-DRB1 *05 (OR: 0.18; *P* = 0.006) and *15 (OR: 0.23; *P* = 0.013) were associated with a reduced risk of hypersensitivity among patients receiving antiretroviral therapy.

**Conclusions:**

The findings of this meta-analysis indicated patients carrying HLA-A *24, HLA-B *18, *35, *39, *51, *81, HLA-C *04 were associated with a higher risk of hypersensitivity. Conversely, subjects carrying HLA-B *15, HLA-C *02, *03, *07, HLA-DRB1 *05, *15 were associated with a reduced risk of hypersensitivity.

**Electronic supplementary material:**

The online version of this article (10.1186/s12879-019-4227-5) contains supplementary material, which is available to authorized users.

## Background

Human leukocyte antigen (HLA) alleles are implicated in drug-induced hypersensitivity, such as that induced by carbamazepine, allopurinol, and phenytoin. Studies shows HLA-B*57:01 are correlated with several adverse drug reactions, ranged from hypersensitivity induced by HIV reverse transcriptase inhibitor guanosine analog abacavir to drug-induced liver injury caused by the penicillin β-lactam antibiotic flucloxacillin or the antitumor tyrosine kinase inhibitor pazopanib [1, 2]. Moreover, nevirapine is widely used for HIV infection combined with other antiretroviral agents [3, 4]. The incidence of hypersensitivity reactions in patients with HIV prescribed abacavir and nevirapine was 5–8% and 6–10%, respectively [2, 5, 6]. The clinical manifestations of these hypersensitivity reactions include fever, rash, fatigue, and gastrointestinal and respiratory symptoms.

As the relationship between HLA polymorphisms and hypersensitivity might affect treatment strategy, HLA allele screening should be conducted in patients with HIV before antiretroviral therapy is initiated [7]. However, data on the alleles of HLA polymorphisms on antiretroviral therapy-induced hypersensitivity are limited and inconclusive. Jesson et al. conducted a systematic review and meta-analysis based on nine studies to evaluate adverse drug reactions (ADRs) among children and adolescent patients treated with abacavir-based regimens. They suggested that the use of abacavir as first- or second-line therapy is associated with improved safety profiles in children and adolescents, whereas the safety profiles of abacavir therapy in relation to HLA alleles were not illustrated [8]. Tangamornsuksan et al. conducted a meta-analysis to evaluate the role of HLA-B*5701 on abacavir-induced hypersensitivity reactions, and found a strong association between HLA-B*5701 and abacavir-induced hypersensitivity. The strength of this relationship led to screening for the HLA-B*5701 allele before initiating abacavir therapy [9]. However, the variety of alleles in HLA has not been illustrated. Numerous studies have already indicated that different HLA alleles may affect the risk of antiretroviral therapy-induced hypersensitivity, whereas the results for individual alleles are inconsistent. The clarification of any potential effect of HLA polymorphisms is particularly important in patients with HIV, as they have not been previously determined. Here, we performed a meta-analysis based on available observational studies to determine the role of HLA polymorphisms on hypersensitivity in patients with HIV administered nevirapine or abacavir therapy.

## Methods

### Data sources, search strategy, and selection criteria

This review was conducted and reported in accordance with the Preferred Reporting Items for Systematic Reviews and Meta-Analysis Statement issued in 2009 (Additional file 1: PRISMA Checklist) [10]. Any study that evaluated the association of various HLA alleles with the risk of nevirapine- or abacavir-induced hypersensitivity in patients with HIV was eligible for inclusion in this meta-analysis, and the selection of studies was not restricted by language or publication status. We searched the PubMed, Embase, Web of Science, and Cochrane Library electronic databases for articles published in April 2019 and used (“Human leukocyte antigen” OR HLA) and (“nevirapine” OR “abacavir” OR “reverse transcriptase inhibitor”) and (“adverse drug reaction” OR “adverse event” OR “hypersensitivity”) as the search terms. The details of the search strategy in PubMed database are presented in Additional file 2: Table S1. We also a manually curated the reference lists from all relevant original and review articles to identify additional eligible studies.

The literature search was independently conducted by two authors using a standardized approach. Any inconsistencies between these two authors were settled by the primary author until a consensus was reached. Studies were included if the following criteria were met: (1) the study had an observational design; (2) all patients were HIV positive; (3) the patients received nevirapine or abacavir therapy; (4) the study regarded HLA polymorphisms as an exposure; and (5) the study reported various alleles of HLA polymorphisms on antiretroviral therapy-induced hypersensitivity. The exclusion criteria were: (1) duplicate studies; (2) the study type was a review, case report, letter, or comments; (3) required data were missing; and (4) the study reported the same populations.

### Data collection and quality assessment

Data collection and quality assessment were carried out by two authors; any inconsistencies between these two authors were settled by group discussion until a consensus was reached. The data collected included the first author’s name, study design, publication year, country, ethnicity, specific hypersensitivity events, number of cases and controls, typing method, mean age, the number of men and women, intervention, and investigated outcomes. The Newcastle-Ottawa Scale (NOS) was used for the evaluation of the quality of observational studies in the meta-analysis, which was based on selection (four items), comparability (one item), and outcome (three items). A “star system” (range, 0–9) has been developed for assessment [11].

### Statistical analysis

The odds ratios (ORs) and corresponding 95% confidence intervals (CIs) were employed to calculate the potential role of four HLA genotypes on the risk of hypersensitivity in patients with HIV using nevirapine or abacavir therapy. The summary ORs and 95%CIs were calculated using a random-effects model due to it assume the true underlying effect varies among included studies [12, 13]. Heterogeneity among the included studies was calculated by using the I^2^ and Q statistics, and a *P* value below 0.10 was considered to indicate significant heterogeneity [14, 15]. Sensitivity analyses were performed to evaluate the effects of a single study from overall analysis according to the intervention type [16]. Moreover, subgroup analyses were conducted for HLA genotypes reported ≥6 cohorts based on country and drugs. Publication biases were calculated if the included subsets had more than six studies using the Egger and Begg tests [17, 18]. The *P* values for summary results were two-sided; with *P* values of < 0.05 considered statistically significant. All statistical analyses were computed by using STATA software (version 10.0; Stata Corporation, College Station, TX, USA).

## Results

**Fig. 1:**
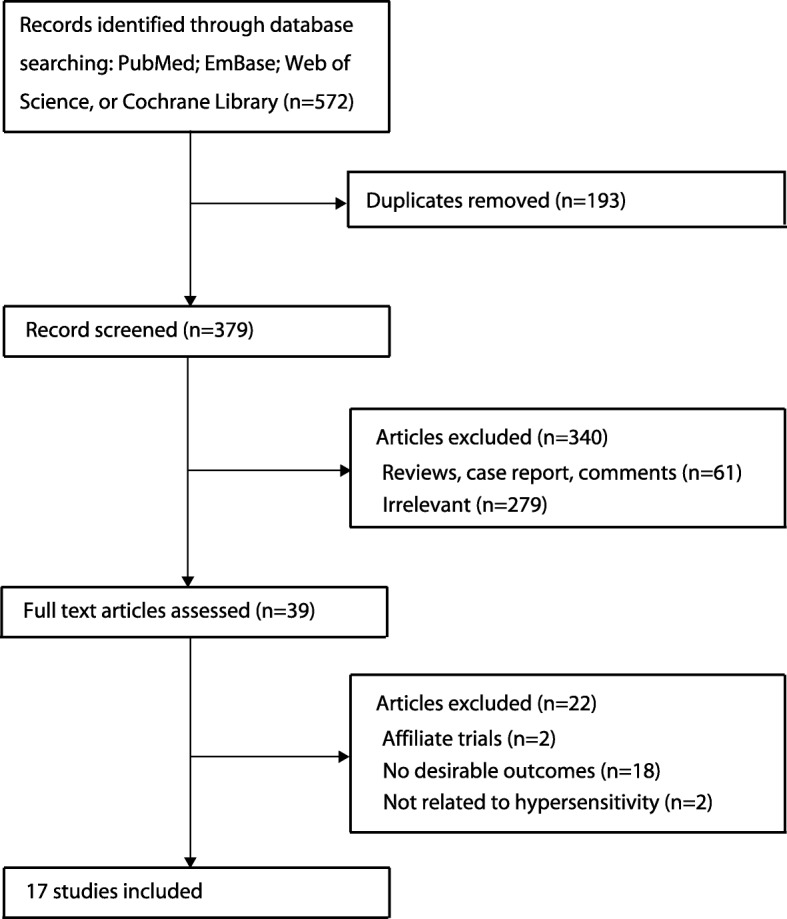
Flow diagram of the literature search and trial selection process

A total of 572 citations from the electronic databases were identified, and 193 duplicate records were excluded. The titles and abstracts were screened for relevant studies, with 340 records being excluded owing to irrelevant topics or because they were reviews, case reports, or comments. Thirty-nine studies were selected for full-text assessment, of which 22 studies were excluded; a total of 17 studies were used for the meta-analysis and quantitative analysis [19–35] (Fig. 1). A manual search of the reference lists of these studies did not yield any new eligible studies. The present meta-analysis included 12 cohort studies and five case-control studies: six studies from Asia; three from Europe; three from Africa; two from the United States; two from Australia, and one from multiple countries. The general characteristics of the included studies are presented in Table 1. The assessment of study quality using NOS revealed five studies with a score of 8, 9 studies with a score of 7, and three studies with a score of 6.

**Table 1:** Baseline characteristics of the studies included in the systematic review and meta-analysis

Study	Study design	Publication year	Country	Ethnicity	Definition of hypersensitivity	Cases	Control	Typing method	Mean age (years)	Men/women	Intervention	NOS score
Littera [19]	Cohort	2006	Italy	Sardinian	extensive skin rash, vesicular, bullous or scaling skin lesions or skin manifestations combined with one or more of the following symptoms: fever (> 38oC),myalgia, arthralgia, visceral impairment, liver toxicity.	13	36	PCR-SSP	38.0	30/19	Nevirapine	7
Chantarangsu [20]	Case control	2009	Thailand	Thai	skin-rash (The skin reactions range from mild localized maculopapular rash, with increasing severity, to diffuse maculopapular rashes, and generalized bullous lesions. Severe fatal reactions, Stevens–Johnson syndrome, and toxic epidermal necrolysis were also observed)	147	185	SBT	35.9	157/175	Nevirapine	8
Gao [21]	Cohort	2012	China	Chinese	extensive skin rash,vesicular, bullous skin lesions, or skin manifestations combined with one or more of the following symptoms: fever (> 38 °C) or liver toxicity.	32	71	PCR-SSP	40.3	41/62	Nevirapine	8
Munderi [22]	Cohort	2011	Uganda	Kampala	fever, rash, gastrointestinal and / or constitutional symptoms and less frequently, respiratory symptoms, lethargy or malaise and usually appearing within 6 weeks of initiating abacavir (median onset 11 days)	6	241	high-resolution DNA- based assay methods	NA	NA	Abacavir	6
Mallal [23]	Cohort	2008	19 countries	mixed	fever, rash, constitutional symptoms, gastrointestinal tract symptoms, and respiratory symptoms that become more severe with continued dosing	66	781	DNA-sequence–based typing	42.0	602/245	Abacavir	8
Yuan [24]	Case control	2011	USA	mixed	Severe cutaneous toxicity [grade III or IV categorized by National Institute of Allergy and Infectious Disease Division of AIDS criteria]; symptomatic grade ≥ 3 hepatic transaminase elevation [alanine transaminase or aspartate aminotransferase > 5× upper limit of normal]; or acute hepatic failure	175	587	Illumina BeadArray technology	42.4	459/303	Nevirapine	8
Pavlos [25]	Case control	2017	USA	USA	Severe cutaneous toxicity [grade III or IV categorized by National Institute of Allergy and Infectious Disease Division of AIDS criteria]; symptomatic grade ≥ 3 hepatic transaminase elevation [alanine transaminase or aspartate aminotransferase > 5× upper limit of normal]; or acute hepatic failure	151	413	PCR amplified	> 18	NA	Nevirapine	7
Carr [26]	Cohort	2013	Malawi	Malawian	widespread rash and systemic manifestations such as fever, cough, or abnormal liver function tests	117	155	SBT	NA	NA	Nevirapine	7
Martin [27]	Cohort	2005	Australia	mixed	fever, hepatitis or rash.	14	221	Serological and SBT or PCR-SS	NA	NA	Nevirapine	7
Vitezica [28]	Cohort	2008	France	White	rash. No liver toxicity was boserved.	6	15	PCR-SSP	NA	NA	Nevirapine	6
Umapathy [29]	Case control	2011	India	Indian	fever, hepatitis, skin rash with clinical complications	40	40	serological	NA	NA	Nevirapine	7
Phillips [30]	Cohort	2013	South Africa	mixed	fever, skin rash and/or hepatitis	57	111	SBT	32.0	55/113	Nevirapine	7
Likanonsakul [31]	Case control	2009	Thailand	Thai	rash	39	60	PCR-SSP	38.4	53/46	Nevirapine	7
Keane [32]	Cohort	2014	Australia	mixed	fever, and/or rash and/or hepatitis	19	262	Serological and SBT or PCR-SS	40.3	247/34	Nevirapine	8
Gatanaga [33]	Cohort	2007	Japan	Japanese	extensive skin rash (accompanied by fever > 38 °C in three) and one patient with chronic hepatitis C who developed nevirapine-induced hepatotoxicity with aspartate aminotransferase/ alanine aminotransferase values three times above the baseline	12	29	PCR-SSP	NA	NA	Nevirapine	6
Gozalo [34]	Cohort	2011	France	mixed	either grade 3 or 4 cutaneous toxicity or alanine aminotransferase elevation greater than three times the upper limit of normal and without other causes being identified.	12	60	PCR-SSO	36.0	53/19	Nevirapine	7
Manglani [35]	Cohort	2018	India	Indian	fever, rash, nausea and vomiting	11	89	PCR-SSP	11.0	61/39	Abacavir	7

The summary results for the relationship between HLA-A alleles and the risk of antiretroviral therapy-induced hypersensitivity are presented in Additional file 3: Table S2. Overall, we noted that patients carrying HLA-A *24 were associated with an increased risk of hypersensitivity (OR: 12.12; 95%CI: 1.53–96.04; *P* = 0.018), whereas the two single nucleotide polymorphisms (SNPs) of HLA-A genotypes *33 (OR: 2.92; 95%CI: 0.39–21.86; *P* = 0.298) and *68 (OR: 0.11; 95%CI: 0.01–1.86; *P* = 0.124) were not associated with the risk of hypersensitivity.

**Fig. 2:**
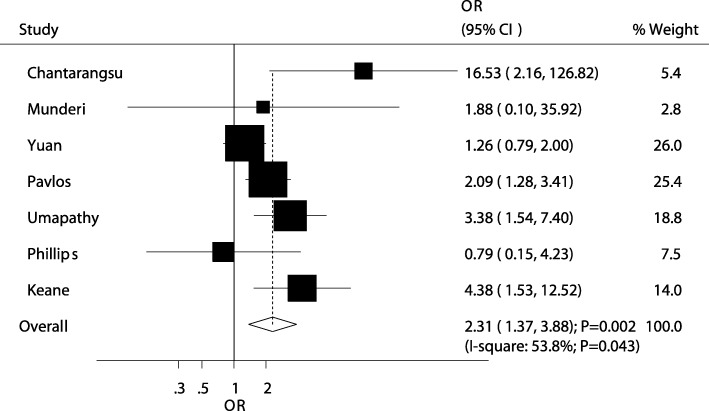
The relationship between HLA-B *35 and the risk of antiretroviral therapy-induced hypersensitivity

A summary of the relationship between HLA-B alleles and the risk of antiretroviral therapy-induced hypersensitivity is presented in Additional file 4: Table S3. First, HLA-B *35 was associated with greater risk of hypersensitivity (OR: 2.31; 95%CI: 1.37–3.88; *P* = 0.002; Fig. 2). A sensitivity analysis indicated that the conclusion was not altered after each study was sequentially excluded (Additional file 5: Table S4); Moreover, subgroup analyses indicated this significant increased risk of hypersensitivity mainly observed in study conducted in Eastern and Western countries, and hypersensitivity induced by nevirapine (Additional file 6: Table S5); furthermore, no significant publication bias was observed (*P* value for Egger: 0.320; P value for Begg: *P* = 0.368; Additional file 7: Figure S1). HLA-B *18 (OR: 1.63; 95%CI: 1.05–2.52; *P* = 0.028), *39 (OR: 11.85; 95%CI: 1.11–125.95; *P* = 0.040), *51 (OR: 1.66; 95%CI: 1.06–2.61; *P* = 0.028), and *81 (OR: 8.11; 95%CI: 1.37–48.13; *P* = 0.021) were correlated with a greater risk of hypersensitivity than the others. Conversely, patients who were carriers of HLA-B *15 had a reduced risk of hypersensitivity (OR: 0.43; 95%CI: 0.27–0.67; *P* < 0.001). Finally, other alleles, including *05, *07, *08, *13, *14, *17, *27, *37, *38, *40, *41, *42, *44, *45, *46, *47, *49, *50, *52, *53, *57, *58, and *82 were not associated with the risk of hypersensitivity.

**Fig. 3:**
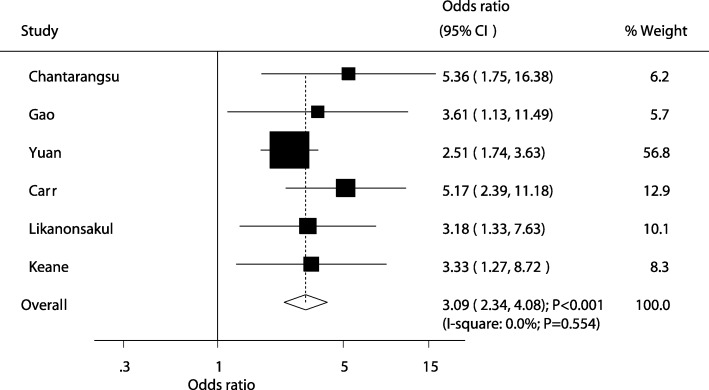
The relationship between HLA-C *04 and the risk of antiretroviral therapy-induced hypersensitivity

A summary of the relationship between HLA-C alleles and the risk of antiretroviral therapy-induced hypersensitivity is presented in Additional file 8: Table S7. We noted HLA-C *04 was associated with an increased risk of hypersensitivity (OR: 3.09; 95%CI: 2.34–4.08; *P* < 0.001; Fig. 3), with no evidence of heterogeneity among the included studies. The results were not affected by the sensitivity analysis (Additional file 5: Table S4). These significant increased the risk of hypersensitivity were detected in all of subsets when stratified by country and drugs (Additional file 6: Table S5). In addition, there was no significant publication bias in the relationship between HLA-C *04 and hypersensitivity risk (*P* value for Egger: 0.071; P value for Begg: *P* = 0.452; Additional file 7: Figure S1). Conversely, patients who were carriers of HLA-C *02 (OR: 0.22; 95%CI: 0.06–0.87; *P* = 0.030), *03 (OR: 0.53; 95%CI: 0.28–1.00; *P* = 0.049), and *07 (OR: 0.61; 95%CI: 0.38–0.99; *P* = 0.044) were associated with a reduced hypersensitivity risk. Finally, there were no significant effects of *01, *05, *06, *08, *12, *14, *15, and *16 in HLA-C on the risk of hypersensitivity.

**Fig. 4:**
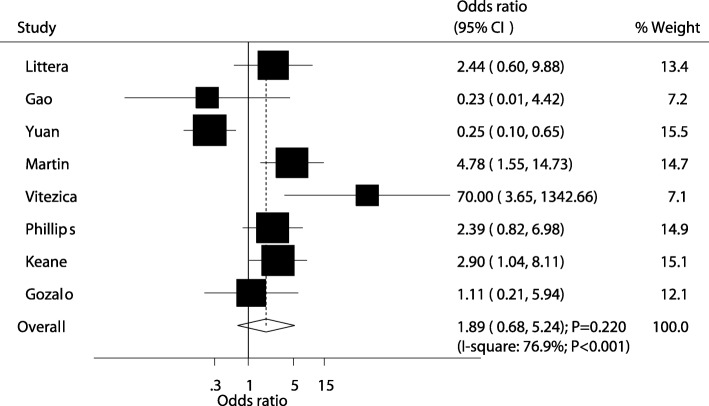
The relationship between HLA-DRB1 *01 and the risk of antiretroviral therapy-induced hypersensitivity

A summary of the relationship between HLA-DRB1 alleles and the risk of antiretroviral therapy-induced hypersensitivity is presented in Additional file 9: Table S8. There was no significant association of HLA-DRB1*01 with the risk of hypersensitivity (OR: 1.89; 95%CI: 0.68–5.24; *P* = 0.220; Fig. 4). Substantial heterogeneity was observed among the included studies (*P* < 0.001), and sensitivity analysis indicated the conclusion was altered by excluding the study conducted by Yuan et al. [24], which specifically associated with higher weight from the overall analysis (Additional file 5: Table S4). The results of subgroup analyses in all subsets were consistent with overall analysis, remained non-significant associations (Additional file 6: Table S5). Further, there was no publication bias for HLA-DRB1*01 and hypersensitivity (*P* value for Egger: 0.526; P value for Begg: *P* = 1.000; Additional file 7: Figure S1). In addition, subjects who were carriers of HLA-DRB1*05 (OR: 0.18; 95%CI: 0.05–0.60; *P* = 0.006) or *15 (OR: 0.23; 95%CI: 0.07–0.74; *P* = 0.013) were associated with a reduced risk of hypersensitivity. Finally, HLA-DRB1 *03, *04, *07, *08, *09, *10, *11, *12, *13, *14, and *16 did not affect the risk of hypersensitivity.

## Discussion

In this study, four HLA polymorphisms of various alleles and the impact on hypersensitivity in patients with HIV treated with nevirapine or abacavir were analyzed. Seventeen studies involving 4273 patients were included in the final analysis. Patients that were carriers of HLA-A *24, HLA-B *18, HLA-B *35, HLA-B *39, HLA-B *51, HLA-B *81, and HLA-C *04 were associated with an increased risk of hypersensitivity. Conversely, HLA-B *15, HLA-C *02, HLA-C *03, HLA-C *07, HLA-DRB1 *05, and HLA-DRB1 *15 were associated with a reduced risk of hypersensitivity in patients administered antiretroviral therapy. No other associations between SNPs of HLA genotypes and the risk of antiretroviral therapy-induced hypersensitivity in HIV patients were detected.

The impact of HLA polymorphisms on hypersensitivity may be mediated through several underlying mechanisms. The Cw*0802 and B*1402 are potentially in strong linkage disequilibrium with specific alleles in genes adjacent to the major histocompatibility complex (MHC) class I region [36]. Furthermore, the substrate(s) of B*3505 and B*1402 might act as triggers of an unfavorable immune response [20]. HLA-DRB1*01:01 elutions directly affected T-cell receptor binding and induced tissue-specific effects on peptides used in antiretroviral therapy.

The relationship between HLA-A polymorphisms and the risk of abacavir-induced hypersensitivity was reported in two studies [22, 23]. A previous study also illustrated that HLA-B*57:01 was associated with an increased risk of abacavir-induced hypersensitivity [37]. However, the effects of other SNPs of HLA polymorphisms on the risk of abacavir-induced hypersensitivity were not calculated. We noted no other correlations of SNPs in HLA-B genotypes with abacavir-induced hypersensitivity. The reason for this was the lower incidence of hypersensitivity, which led to results with broad 95% confidence intervals [22, 23]. Further, the relationship between each SNP of the HLA-A alleles and antiretroviral-induced hypersensitivity was mentioned in one study. The variation of these results may be caused by the broad confidence intervals, resulting in no statistically significant difference. Third, five alleles in the HLA-B genotypes with greater hypersensitivity risk and one allele in HLA-B were correlated with lower hypersensitivity risk. Various alleles in the HLA-B genotypes affected CD8^+^ and CD4^+^ T cells, which play an important role in the immunopathogenesis of antiretroviral-induced hypersensitivity. These results were mainly focused on necirapine-induced hypersensitivity, with just two studies reporting the abacavir-induced hypersensitivity risk. Fourth, several SNPs of HLA-C genotypes showed stronger linkage disequilibrium with necirapine-induced hypersensitivity, and the specific event of hypersensitivity was not consistent among the included studies. Finally, the incidence of necirapine-induced hypersensitivity was lower in the subjects that were carriers of several SNPs of the HLA-DRB1 genotype. These results could be due to the risk of necirapine-induced hypersensitivity, which was independent of ethnicity but associated with different sensitivities to the same allergen; thus, the degree of immune depression varied with ethnicity [38, 39].

The strengths of this systematic review and meta-analysis were the comprehensive literature search and the thorough evaluation of the HLA polymorphisms of the various alleles. The primary limitation was the deficiency of eligible studies. The sample sizes of most selected studies were small. Thus, the relationships between HLA polymorphisms was not sufficiently convincing, and the applicability of the findings was limited. Subgroup analyses just conducted for HLA genotypes reported ≥6 cohorts based on country and drugs and the source of heterogeneity were not fully interpretation owing to the small number of studies for many alleles. Further, although the ethnicity was reported in most studies, the provided data according to ethnicity was not available to extract. Therefore, subgroup analyses just provided based on countries, which might be affected by ethnicity. In addition, specific individual data were unavailable for all the trials, which restricted the analysis at a study level. Moreover, this meta-analysis comprised cohort and case control studies, which might introduce potential uncontrolled confounders. The design of the study and the characteristics of the patients might affect the statistical power. Finally, the definition of hypersensitivity events are not consistent among included studies, which might introduce uncontrolled confounders.

This meta-analysis demonstrated that patients carrying the HLA-A *24, HLA-B *18, HLA-B *35, HLA-B *39, HLA-B *51, HLA-B *81, and HLA-C *04 alleles were associated with increased antiretroviral-induced hypersensitivity risk. In patients with HIV, especially for HLA-B *35 and HLA-C *04, the results were quite stable because of the pooled results. The impact of other SNPs should be verified in large-scale studies. Further, numerous relationships were variable owing to the small number of included studies. Further larger-scale, prospective studies should be conducted to explore these relationships in patients from various races or with specific characteristics to clearly determine the value in clinical practice.

## Conclusions

In summary, patients carrying HLA-A *24, HLA-B *18, *35, *39, *51, *81, HLA-C *04 were correlated with a higher risk (*P* < 0.05) of hypersensitivity. Conversely, subjects carrying HLA-B *15, HLA-C *02, *03, *07, HLA-DRB1 *05, *15 were associated with a reduced risk (*P* < 0.05) of hypersensitivity. Further larger-scale, prospective studies should be conducted to explore these relationships in patients from various races or with specific characteristics to clearly determine the value in clinical practice.

## Additional files

Additional file 1: PRISMA Checklist. (DOC 53 kb)

Additional file 2: **Table S1.** The details of the search strategy in PubMed database. (DOCX 16 kb)

Additional file 3: **Table S2.** The summary results for the relationship between HLA-A and the risk of hypersensitivity. (DOCX 16 kb)

Additional file 4; **Table S3.** The summary results for the relationship between HLA-B and the risk of hypersensitivity. (DOCX 19 kb)

Additional file 5: **Table S4–1.** Sensitivity analysis for HLA-B *35. **Table S4–2.** Sensitivity analysis for HLA-C *04. **Table S4–3.** Sensitivity analysis for HLA-DRB1 *01. (DOCX 19 kb)

Additional file 6: **Table S5.** Subgroup analysis for HLA-B *35, HLA-C *04, and HLA-DRB1 *01. (DOCX 19 kb)

Additional file 7: **Figure S1.** Funnel plot for HLA-B *35. **Figure S2.** Funnel plot for HLA-C *04. **Figure S3.** Funnel plot for HLA-DRB1*01. (DOCX 349 kb)

Additional file 8: **Table S7.** The summary results for the relationship between HLA-C and the risk of hypersensitivity. (DOCX 18 kb)

Additional file 9: **Table S8.** The summary results for the relationship between HLA-DRB1 and the risk of hypersensitivity. (DOCX 18 kb)

## Data Availability

All data generated or analysed during this study are included in this published article and its supplementary information files.

## Abbreviations

ADRs: Adverse drug reactions
CIs: Confidence intervals
HIV: Human immunodeficiency virus
HLA: Human leukocyte antigen
MHC: Major histocompatibility complex
NOS: The Newcastle-Ottawa Scale
ORs: Odds ratios
SNPs: Single nucleotide polymorphisms
