# Supplementary material for: Associations between human leukocyte antigen polymorphisms and hypersensitivity to antiretroviral therapy in patients with human immunodeficiency virus: a meta-analysis
Source: BMC Infect Dis. 2019 Jul 5;19:583. doi: 10.1186/s12879-019-4227-5 (PMC6612203; doi:10.1186/s12879-019-4227-5)
Supplement: Supplementary file 2 — Table S1. The details of the search strategy in PubMed database. (DOCX 16 kb) [file 12879_2019_4227_MOESM2_ESM.docx]

Additional file 2 Table S1. The details of the search strategy in PubMed database

| **#** | **Search term** |
| --- | --- |
| 1 | “Human leukocyte antigen” OR “human leukocyte antigens” OR HLA |
| 2 | “nevirapine” OR“entecavir” OR “lamivudine” OR “adefovir” OR “tenofovir” OR “telbivudine” OR “reverse transcriptase inhibitor” |
| 3 | ADR or "adverse drug reaction" or "adverse drug reactions" or "adverse event" or "adverse events" |
| 4 | "Drug effect" or "drug effects" or "drug toxicity" |
| 5 | “hypersensitivity” |
| 6 | #1 and #2 and (#3 or #4 or #5) |
