# Supplementary material for: Associations between human leukocyte antigen polymorphisms and hypersensitivity to antiretroviral therapy in patients with human immunodeficiency virus: a meta-analysis
Source: BMC Infect Dis. 2019 Jul 5;19:583. doi: 10.1186/s12879-019-4227-5 (PMC6612203; doi:10.1186/s12879-019-4227-5)
Supplement: Supplementary file 3 — Table S2. The summary results for the relationship between HLA-A and the risk of hypersensitivity. (DOCX 16 kb) [file 12879_2019_4227_MOESM3_ESM.docx]

Additional file 3 Table S2. The summary results for the relationship between HLA-A and the risk of hypersensitivity

| **Allele** | **Reference** | **OR and 95% CI** | **P value** | **Heterogeneity（%）** | **P value for heterogeneity** |
| --- | --- | --- | --- | --- | --- |
| *24 | 20 | 12.12 (1.53-96.04) | 0.018 | - | - |
| *33 | 19 | 2.92 (0.39-21.86) | 0.298 | - | - |
| *68 | 30 | 0.11 (0.01-1.86) | 0.124 | - | - |
