# Supplementary material for: Associations between human leukocyte antigen polymorphisms and hypersensitivity to antiretroviral therapy in patients with human immunodeficiency virus: a meta-analysis
Source: BMC Infect Dis. 2019 Jul 5;19:583. doi: 10.1186/s12879-019-4227-5 (PMC6612203; doi:10.1186/s12879-019-4227-5)
Supplement: Supplementary file 4 — Table S3. The summary results for the relationship between HLA-B and the risk of hypersensitivity. (DOCX 19 kb) [file 12879_2019_4227_MOESM4_ESM.docx]

Additional file 4 Table S3. The summary results for the relationship between HLA-B and the risk of hypersensitivity

| **Allele** | **Reference** | **OR and 95% CI** | **P value** | **Heterogeneity（%）** | **P value for heterogeneity** |
| --- | --- | --- | --- | --- | --- |
| *05 | 29 | 1.71 (0.39-7.41) | 0.473 | - | - |
| *07 | 22,25,29,30 | 0.73 (0.28-1.93) | 0.523 | 58.5 | 0.065 |
| *08 | 22, 25, 29 | 0.77 (0.27-2.20) | 0.623 | 49.3 | 0.139 |
| *13 | 22, 25 | 1.17 (0.66-2.08) | 0.598 | 0.0 | 0.798 |
| *14 | 19,22,25, | 1.93 (0.27-14.06) | 0.514 | 75.9 | 0.016 |
| *15 | 22,25,29,30 | 0.43 (0.27-0.67) | <0.001 | 0.0 | 0.501 |
| *17 | 29 | 1.67 (0.68-4.12) | 0.263 | - | - |
| *18 | 22,25,30 | 1.63 (1.05-2.52) | 0.028 | 0.0 | 0.527 |
| *27 | 22,25 | 0.91 (0.26-3.25) | 0.889 | 17.4 | 0.271 |
| *35 | 20,22,24,25,29,30,32 | 2.31 (1.37-3.88) | 0.002 | 53.8 | 0.043 |
| *37 | 22,25,29 | 1.58 (0.25-9.99) | 0.625 | 50.4 | 0.133 |
| *38 | 20,25 | 0.63 (0.10-4.09) | 0.627 | 86.3 | 0.007 |
| *39 | 22 | 11.85 (1.11-125.95) | 0.040 | - | - |
| *40 | 22,29 | 1.31 (0.58-2.95) | 0.512 | 0.0 | 0.445 |
| *41 | 22,30 | 0.63 (0.07-5.91) | 0.687 | 13.3 | 0.283 |
| *42 | 22 | 0.39 (0.02-7.14) | 0.529 | - | - |
| *44 | 22,29 | 0.63 (0.14-2.96) | 0.562 | 0.0 | 0.939 |
| *45 | 22 | 2.94 (0.52-16.68) | 0.223 | - | - |
| *46 | 25 | 0.98 (0.49-1.95) | 0.954 | - | - |
| *47 | 22 | 7.83 (0.79-77.73) | 0.079 | - | - |
| *49 | 22 | 0.99 (0.05-18.25) | 0.993 | - | - |
| *50 | 22 | 12.33 (0.46-332.41) | 0.135 | - | - |
| *51 | 22,25 | 1.66 (1.06-2.61) | 0.028 | 0.0 | 0.987 |
| *52 | 25 | 0.19 (0.03-1.48) | 0.095 | - | - |
| *53 | 22,26 | 1.78 (0.11-28.80) | 0.686 | 71.8 | 0.060 |
| *57 | 22,23,25 | 5.37 (0.38-76.73) | 0.215 | 96.0 | <0.001 |
| *58 | 22,30 | 1.02 (0.52-1.98) | 0.959 | 0.0 | 0.535 |
| *81 | 22 | 8.11 (1.37-48.13) | 0.021 | - | - |
| *82 | 22 | 3.31 (0.16-66.31) | 0.434 | - | - |
