# Supplementary material for: Associations between human leukocyte antigen polymorphisms and hypersensitivity to antiretroviral therapy in patients with human immunodeficiency virus: a meta-analysis
Source: BMC Infect Dis. 2019 Jul 5;19:583. doi: 10.1186/s12879-019-4227-5 (PMC6612203; doi:10.1186/s12879-019-4227-5)
Supplement: Supplementary file 5 — Table S4–1. Sensitivity analysis for HLA-B *35. Table S4–2. Sensitivity analysis for HLA-C *04. Table S4–3. Sensitivity analysis for HLA-DRB1 *01. (DOCX 19 kb) [file 12879_2019_4227_MOESM5_ESM.docx]

Additional file 5 Table S4-1. Sensitivity analysis for HLA-B *35

| Excluding study | OR and 95%CI | P value | Heterogeneity (%) | P value for heterogeneity |
| --- | --- | --- | --- | --- |
| Chantarangsu | 2.03 (1.29-3.18) | 0.002 | 42.5 | 0.122 |
| Munderi | 2.35 (1.35-4.08) | 0.002 | 61.5 | 0.023 |
| Yuan | 2.81 (1.64-4.79) | <0.001 | 31.6 | 0.199 |
| Pavlos | 2.51 (1.20-5.29) | 0.015 | 61.2 | 0.024 |
| Umapathy | 2.13 (1.18-3.84) | 0.012 | 54.0 | 0.054 |
| Phillips | 2.52 (1.46-4.35) | 0.001 | 57.7 | 0.037 |
| Keane | 2.07 (1.20-3.57) | 0.009 | 52.7 | 0.061 |

Table S4-2. Sensitivity analysis for HLA-C *04

| Excluding study | OR and 95%CI | P value | Heterogeneity (%) | P value for heterogeneity |
| --- | --- | --- | --- | --- |
| Chantarangsu | 2.98 (2.24-3.97) | <0.001 | 0.0 | 0.562 |
| Gao | 3.06 (2.30-4.07) | <0.001 | 0.0 | 0.421 |
| Yuan | 4.07 (2.67-6.20) | <0.001 | 0.0 | 0.892 |
| Carr | 2.86 (2.13-3.85) | <0.001 | 0.0 | 0.735 |
| Likanonsakul | 3.08 (2.30-4.13) | <0.001 | 0.0 | 0.411 |
| Keane | 3.07 (2.30-4.10) | <0.001 | 0.0 | 0.414 |

Table S4-3. Sensitivity analysis for HLA-DRB1 *01

| Excluding study | OR and 95%CI | P value | Heterogeneity (%) | P value for heterogeneity |
| --- | --- | --- | --- | --- |
| Littera | 1.84 (0.59-5.77) | 0.297 | 79.0 | <0.001 |
| Gao | 2.21 (0.78-6.21) | 0.134 | 78.1 | <0.001 |
| Yuan | 2.77 (1.39-5.52) | 0.004 | 35.6 | 0.156 |
| Martin | 1.62 (0.54-4.87) | 0.392 | 75.6 | <0.001 |
| Vitezica | 1.46 (0.57-3.74) | 0.432 | 73.3 | 0.001 |
| Phillips | 1.85 (0.56-6.07) | 0.313 | 78.8 | <0.001 |
| Keane | 1.78 (0.55-5.7) | 0.338 | 78.0 | <0.001 |
| Gozalo | 2.05 (0.67-6.31) | 0.208 | 79.1 | <0.001 |
