# Supplementary material for: Associations between human leukocyte antigen polymorphisms and hypersensitivity to antiretroviral therapy in patients with human immunodeficiency virus: a meta-analysis
Source: BMC Infect Dis. 2019 Jul 5;19:583. doi: 10.1186/s12879-019-4227-5 (PMC6612203; doi:10.1186/s12879-019-4227-5)
Supplement: Supplementary file 6 — Table S5. Subgroup analysis for HLA-B *35, HLA-C *04, and HLA-DRB1 *01. (DOCX 19 kb) [file 12879_2019_4227_MOESM6_ESM.docx]

Additional file 6 Table S5. Subgroup analysis for HLA-B *35, HLA-C *04, and HLA-DRB1 *01

| HLA polymorphisms | Group | OR and 95% CI | P value | Heterogeneity (%) | P value for heterogeneity |
| --- | --- | --- | --- | --- | --- |
| HLA-B *35 | Country | | | | |
|  | Eastern | 5.60 (1.31-23.82) | 0.020 | 50.8 | 0.154 |
|  | Western | 1.96 (1.10-3.49) | 0.022 | 62.6 | 0.069 |
|  | Africa | 0.98 (0.23-4.17) | 0.974 | 0.0 | 0.615 |
|  | Drugs | | | | |
|  | Nevirapine | 2.35 (1.35-4.08) | 0.002 | 61.5 | 0.023 |
|  | Abacavir | 1.88 (0.10-35.63) | 0.674 | - | - |
| HLA-C *04 | Country | | | | |
|  | Eastern | 3.8 (2.10-6.88) | <0.001 | 0.0 | 0.767 |
|  | Western | 2.60 (1.85-3.67) | <0.001 | 0.0 | 0.591 |
|  | Africa | 5.17 (2.39-11.18) | <0.001 | - | - |
|  | Drugs | | | | |
|  | Nevirapine | 3.09 (2.34-4.08) | <0.001 | 0.0 | 0.554 |
|  | Abacavir | - | - | - | - |
| HLA-DRB1 *01 | Country | | | | |
|  | Eastern | 0.23 (0.01-4.84) | 0.344 | - | - |
|  | Western | 2.26 (0.64-7.92) | 0.204 | 81.4 | <0.001 |
|  | Africa | 2.39 (0.82-6.97) | 0.111 | - | - |
|  | Drugs | | | | |
|  | Nevirapine | 1.89 (0.70-5.13) | 0.210 | 75.8 | <0.001 |
|  | Abacavir | - | - | - | - |
