# Supplementary figures and images for: Associations between human leukocyte antigen polymorphisms and hypersensitivity to antiretroviral therapy in patients with human immunodeficiency virus: a meta-analysis
Source: BMC Infect Dis. 2019 Jul 5;19:583. doi: 10.1186/s12879-019-4227-5 (PMC6612203; doi:10.1186/s12879-019-4227-5)

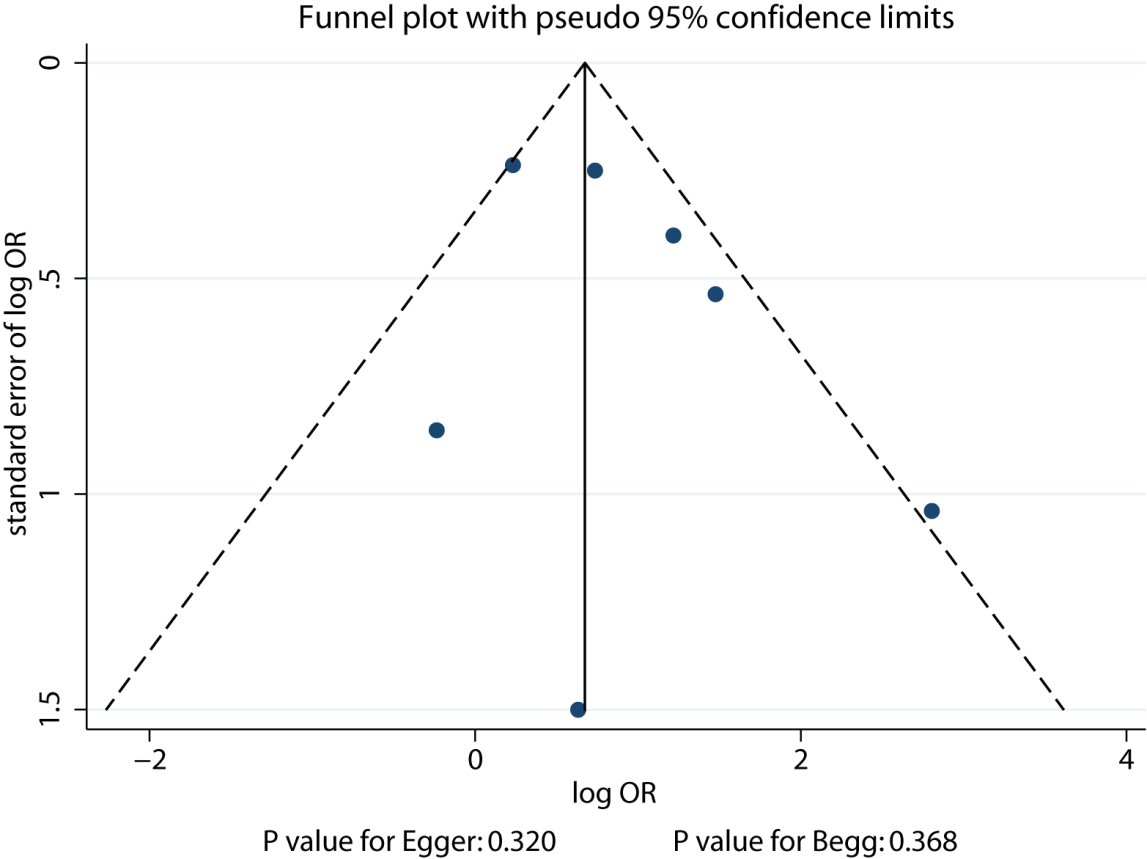


Figure S1. Funnel plot for HLA-B *35


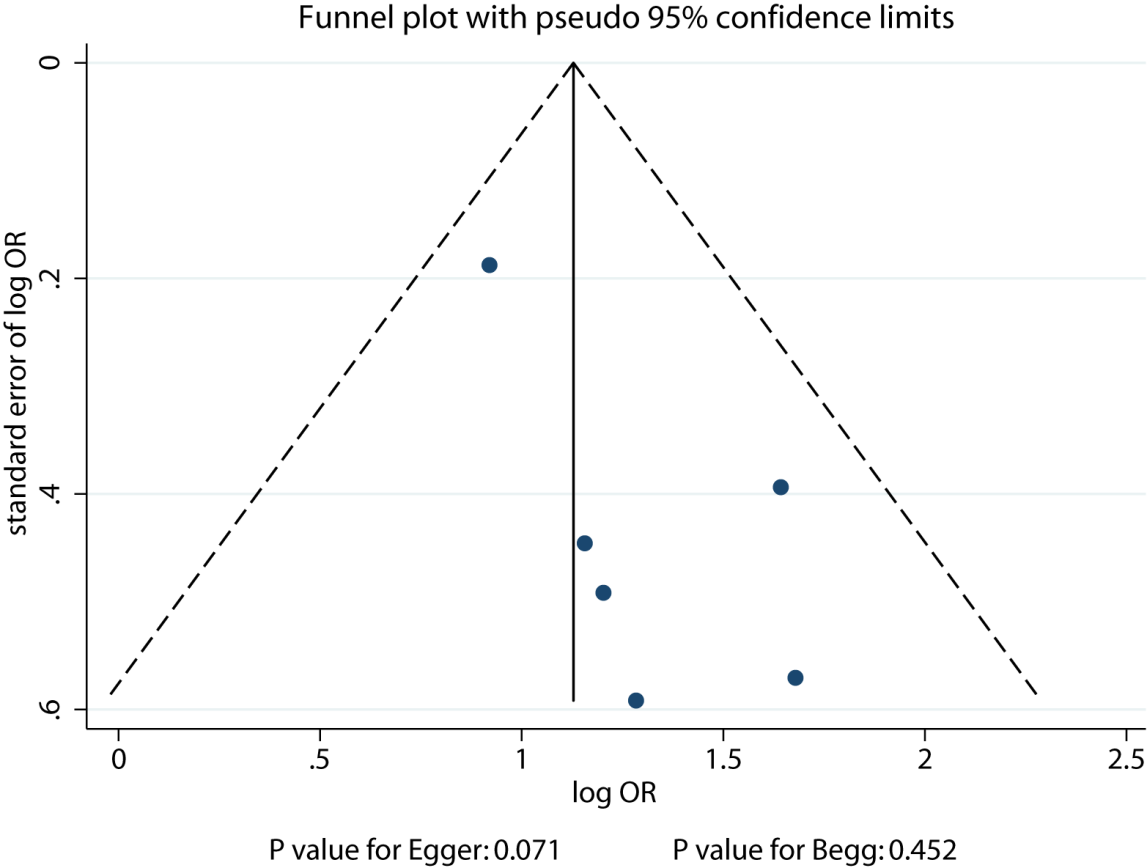


Figure S2. Funnel plot for HLA-C *04


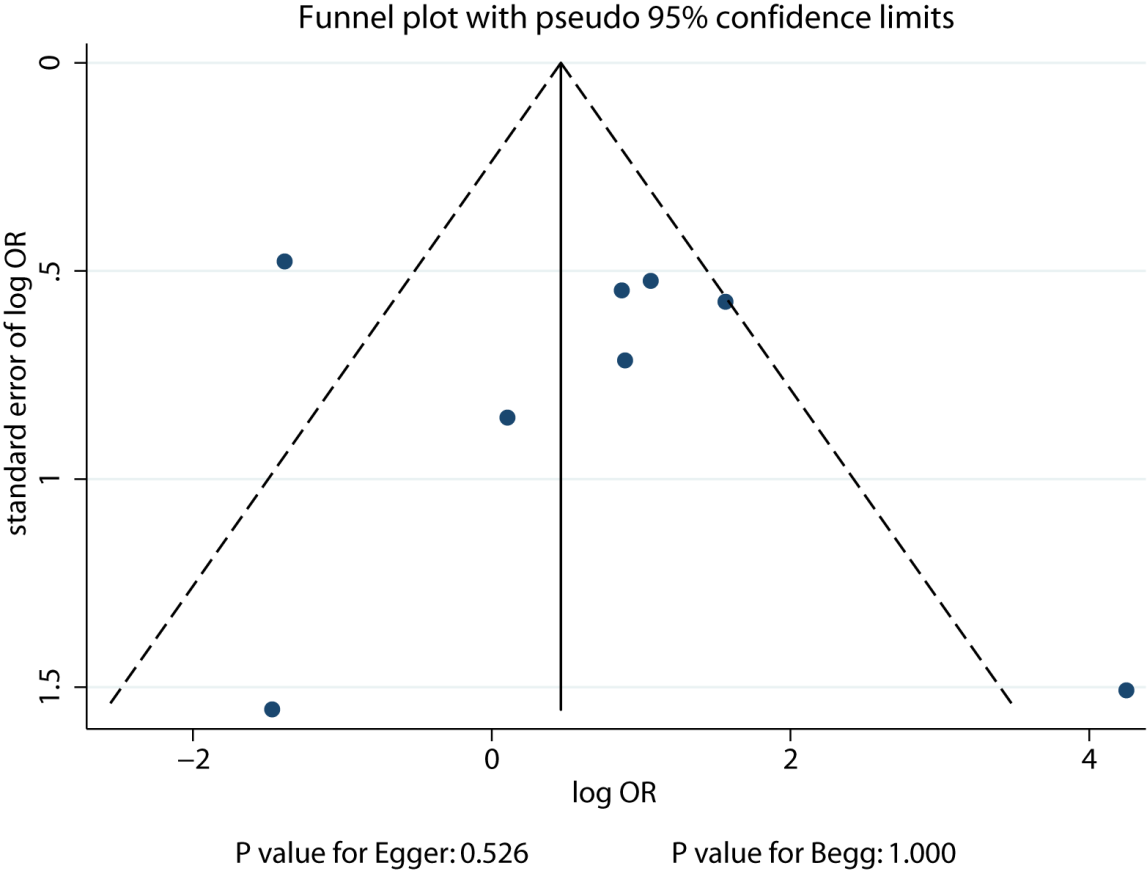


Figure S3. Funnel plot for HLA-DRB1*01

Supplement: Supplementary file 7 — Figure S1. Funnel plot for HLA-B *35. Figure S2. Funnel plot for HLA-C *04. Figure S3. Funnel plot for HLA-DRB1*01. (DOCX 349 kb) [file 12879_2019_4227_MOESM7_ESM.docx]
