# Supplementary material for: Associations between human leukocyte antigen polymorphisms and hypersensitivity to antiretroviral therapy in patients with human immunodeficiency virus: a meta-analysis
Source: BMC Infect Dis. 2019 Jul 5;19:583. doi: 10.1186/s12879-019-4227-5 (PMC6612203; doi:10.1186/s12879-019-4227-5)
Supplement: Supplementary file 8 — Table S7. The summary results for the relationship between HLA-C and the risk of hypersensitivity. (DOCX 18 kb) [file 12879_2019_4227_MOESM8_ESM.docx]

Additional file 8 Table S7. The summary results for the relationship between HLA-C and the risk of hypersensitivity

| **Allele** | **Number of studies** | **OR and 95% CI** | **P value** | **Heterogeneity（%）** | **P value for heterogeneity** |
| --- | --- | --- | --- | --- | --- |
| *01 | 21,31 | 1.16 (0.60-2.25) | 0.652 | 16.9 | 0.273 |
| *02 | 21,30 | 0.22 (0.06-0.87) | 0.030 | 0.0 | 0.428 |
| *03 | 21,30,31 | 0.53 (0.28-1.00) | 0.049 | 24.5 | 0.266 |
| *04 | 20,21,24,26,31,32 | 3.09 (2.34-4.08) | <0.001 | 0.0 | 0.554 |
| *05 | 21,31 | 0.17 (0.00-11.86) | 0.414 | 84.3 | 0.012 |
| *06 | 21,30,31 | 0.76 (0.38-1.52) | 0.441 | 30.9 | 0.235 |
| *07 | 20,21,31 | 0.61 (0.38-0.99) | 0.044 | 27.5 | 0.252 |
| *08 | 19,21,31,32,33 | 2.39 (0.97-5.93) | 0.059 | 61.5 | 0.034 |
| *12 | 21,30,31 | 1.48 (0.47-4.64) | 0.501 | 56.6 | 0.100 |
| *14 | 21,31 | 0.99 (0.13-7.55) | 0.992 | 48.5 | 0.163 |
| *15 | 21,31 | 1.53 (0.40-5.89) | 0.536 | 0.0 | 0.984 |
| *16 | 21 | 6.81 (0.27-171.79) | 0.244 | - | - |
