# Supplementary material for: Associations between human leukocyte antigen polymorphisms and hypersensitivity to antiretroviral therapy in patients with human immunodeficiency virus: a meta-analysis
Source: BMC Infect Dis. 2019 Jul 5;19:583. doi: 10.1186/s12879-019-4227-5 (PMC6612203; doi:10.1186/s12879-019-4227-5)
Supplement: Supplementary file 9 — Table S8. The summary results for the relationship between HLA-DRB1 and the risk of hypersensitivity. (DOCX 18 kb) [file 12879_2019_4227_MOESM9_ESM.docx]

Additional file 9 Table S8. The summary results for the relationship between HLA-DRB1 and the risk of hypersensitivity

| **Allele** | **Number of studies** | **OR and 95% CI** | **P value** | **Heterogeneity（%）** | **P value for heterogeneity** |
| --- | --- | --- | --- | --- | --- |
| *01 | 19,21,24,27,28,30,32,34 | 1.89 (0.68-5.24) | 0.220 | 76.9 | <0.001 |
| *03 | 21,30 | 0.49 (0.19-1.25) | 0.134 | 0.0 | 0.912 |
| *04 | 21 | 1.71 (0.58-4.99) | 0.328 | - | - |
| *05 | 26 | 0.18 (0.05-0.60) | 0.006 | - | - |
| *07 | 21 | 0.98 (0.28-3.47) | 0.980 | - | - |
| *08 | 21 | 1.05 (0.38-2.88) | 0.932 | - | - |
| *09 | 21 | 0.96 (0.42-2.22) | 0.924 | - | - |
| *10 | 21 | 2.26 (0.14-37.28) | 0.569 | - | - |
| *11 | 21 | 2.01 (0.56-7.14) | 0.282 | - | - |
| *12 | 20,21 | 1.87 (0.87-4.01) | 0.108 | 49.4 | 0.160 |
| *13 | 21 | 1.73 (0.36-8.24) | 0.490 | - | - |
| *14 | 21,30 | 1.06 (0.06-19.03) | 0.968 | 64.7 | 0.092 |
| *15 | 21 | 0.23 (0.07-0.74) | 0.013 | - | - |
| *16 | 21 | 2.39 (0.56-10.25) | 0.240 | - | - |
